# Older people’s attitudes towards deprescribing cardiometabolic medication

**DOI:** 10.1186/s12877-021-02249-z

**Published:** 2021-06-16

**Authors:** Stijn Crutzen, Jamila Abou, Sanne E. Smits, Gert Baas, Jacqueline G. Hugtenburg, Mette Heringa, Petra Denig, Katja Taxis

**Affiliations:** 1 Department of Clinical Pharmacy and Pharmacology, University Medical Center Groningen, University of Groningen, Groningen, Netherlands; 2 Universitair Medisch Centrum Groningen, Petra Denig Clinical Pharmacy and Pharmacology, EB70, Postbus 30.001, Hanzeplein1, 9700 RB Groningen, The Netherlands; 3 Department of Clinical Pharmacology and Pharmacy, Amsterdam UMC, location VUMC, Amsterdam, The Netherlands; 4 SIR Institute for Pharmacy Practice and Policy, Theda Mansholtstraat 5B, 2331 JE Leiden, The Netherlands; 5 Division of Pharmacoepidemiology and Clinical Pharmacology, Utrecht Institute for Pharmaceutical Sciences, Utrecht University, Utrecht, The Netherlands; 6 Unit of PharmacoTherapy, Epidemiology and Economics, Groningen Research Institute of Pharmacy, University of Groningen, Groningen, The Netherlands

**Keywords:** Deprescribing, Aged, Cardiometabolic medication, Survey

## Abstract

**Background:**

Overtreatment with cardiometabolic medication in older patients can lead to major adverse events. Timely deprescribing of these medications is therefore essential. Self-reported willingness to stop medication is usually high among older people, still overtreatment with cardiometabolic medication is common and deprescribing is rarely initiated. An important barrier for deprescribing reported by general practitioners is the patients’ unwillingness to stop the medication. More insights are needed into the influence of patients’ characteristics on their attitudes towards deprescribing and differences in these attitudes between cardiometabolic medication groups.

**Methods:**

A survey in older people using cardiometabolic medication using the revised Patients’ Attitudes Towards Deprescribing (rPATD) questionnaire was performed. Participants completed the general rPATD and an adapted version for four medication groups. Linear and ordinal logistic regression were used to assess the influence of age, sex, therapeutic area and number of medications used on the patients’ general attitudes towards deprescribing. Univariate analysis was used to compare differences in deprescribing attitudes towards sulfonylureas, insulins, antihypertensive medication and statins.

**Results:**

Overall, 314 out of 1143 invited participants completed the survey (median age 76 years, 54% female). Most participants (80%) were satisfied with their medication and willing to stop medications if their doctor said it was possible (88%). Age, sex and therapeutic area had no influence on the general attitudes towards deprescribing. Taking more than ten medicines was significantly associated with a higher perceived medication burden. Antihypertensive medication and insulin were considered more appropriate than statins, and insulin was considered more appropriate than sulfonylureas not favouring deprescribing.

**Conclusions:**

The majority of older people using cardiometabolic medication are willing to stop one of their medicines if their doctor said it was possible. Health care providers should take into account that patients perceive some of their medication as more appropriate than other medication when discussing deprescribing.

**Supplementary Information:**

The online version contains supplementary material available at 10.1186/s12877-021-02249-z.

## Background

Intensive control of lipids, glucose and blood pressure is essential in the management of type 2 diabetes and cardiovascular diseases in order to decrease long-term risks of complications [1–3]. In an older population the benefits of intensive treatment with cardiometabolic medication is a subject of debate [4–8]. Overtreatment with cardiometabolic medication can lead to hospitalisation due to major adverse events like falls and hypoglycaemia [9–11]. Additionally, there are indications that the benefits of intensive treatment have been overestimated for older patients [12, 13]. Timely deprescribing of cardiometabolic medication is therefore relevant in an older population [14, 15]. Deprescribing is the planned process in which medication is reduced or stopped by a health care provider, in consultation with the patient, to improve patient outcomes [16, 17]. Research about the effects of deprescribing cardiometabolic medication is scarce but it seems feasible without unacceptable deterioration in glycaemic or blood pressure control [18–20]. Involving patients in the process of deprescribing is important to improve satisfaction, adherence, well-being and health outcomes, and a majority of older patients want to be involved in decision making about their medication [21–23]. Despite such findings, overtreatment is still very common and deprescribing seems to be rarely initiated in people with type 2 diabetes and cardiovascular diseases [9, 24–26].

In survey studies, self-reported willingness to stop medication is common among older people, with 70 to 90% of patients willing to stop one or more medicines if proposed by their doctor [21, 22, 27–36]. Paradoxically, around 90% of these patients also report being satisfied with their current medication [21, 30]. Patients’ resistance towards stopping is often considered to be a barrier for deprescribing by physicians [37–39]. Patients themselves also mention several barriers to stopping medication. Previous qualitative research showed that poor experiences with stopping and the belief that the medication is still needed can be major barriers for patients [39–42]. Furthermore, patients do not always see the need to stop medication they have been taking for a long time and medication they experience no harm from [32, 40]. For cardiometabolic medication in particular, confusion about changing treatment targets, uncertainty about the risks and benefits, and inconsistent feelings towards deprescribing were identified as barriers [43, 44].

Studies performed in the United States (US) and Malaysia showed that some patient characteristics like age, sex, education and number of medications were associated with attitudes towards deprescribing [30, 45]. Older age was associated with attitudes towards deprescribing among Malaysian patients aged 60 years and older [30] but not among Swiss patients aged 70 years and older [27]. The number of medications was associated with some of the attitudes favoring deprescribing [30]. The use of potentially inappropriate medication did not seem to be associated with the willingness to deprescribe [28]. So far, studies have not investigated differences in attitudes of patients using different kinds of preventive medications, such as diabetes or cardiovascular medication. Patients may perceive some of their medications as more important than others. This depends on the disease for which the medication is prescribed, side effects, the perceived effectiveness of the medication and whether or not the effects of the medication can be observed through for instance laboratory test results [46, 47]. More insight in the differences in attitudes towards deprescribing between patients based on commonly available demographic and treatment characteristics may be helpful for health care providers in tailoring their conversations with patients about deprescribing.

Our aim was to elucidate whether age, sex, therapeutic area, and total number of medications used were associated with the patients’ general attitudes towards deprescribing. Secondly, we studied whether there were differences in perceived appropriateness and concerns towards stopping of different cardiometabolic medication groups.

## Methods

### Study design and participants

A survey study was performed using the linguistically validated Dutch version of the revised Patients’ Attitudes Towards Deprescribing (rPATD) questionnaire [48, 49]. In addition to the rPATD questionnaire, an adapted version of the rPATD was included which was modified to investigate the attitudes towards deprescribing of a specific medication group instead of deprescribing in general, as proposed by Edelman et al. [48]. In this adapted part the items on appropriateness and concerns about stopping medication for four cardiometabolic medication groups were included. No additional validation was done for these specific items. Participants were included who were (1) 70 years or older, (2) were able to read and write in Dutch, and (3a) used a sulfonylurea and/or insulin, or (3b) used a statin and at least one antihypertensive but no insulin or sulfonylurea. The ability to read and write in Dutch was implicitly assumed since only surveys in Dutch were offered. Based on inclusion criteria 3a/3b, participants received specific rPATDs about those medication groups. This resulted in the following groups of patients completing the medication specific rPATD questions for (1) sulfonylurea only, (2) insulin only, (3) sulfonylurea and insulin, (4) antihypertensive medication and statin. No formal sample size calculation was made but using a rule-of-thumb for regression analysis (*N* ≥ 50 + 8 m, where m is the number of determinants) we would need at least 82 participants [50]. A pragmatic recruitment strategy was used aimed at recruiting at least 300 participants through five community pharmacies across the Netherlands. They were identified by the community pharmacist and a researcher using dispensing data from the pharmacy information system. The following Anatomical Therapeutic Chemical (ATC) classification system codes were used to assess medication use: statin (C10AA), antihypertensive medication (C02/C03/C07/C08/C09), sulfonylurea (A10BB) and insulin (A10A) [51]. Potential participants were invited using an email with a link to the online questionnaire (Qualtrics XM), or by mail with a paper version in case no email address was available. No reminders were sent. A question on informed consent was included in the beginning of the questionnaire and was collected from all participants. The online version was password protected and only one survey could be completed per IP address to ensure no duplicate surveys were completed. Participants were unable to skip questions but they could abort the process of completing the questionnaire. Items were not randomised, three rPATD items per page were shown and participants could go back to change their answers. Usability and the technical functionality of the questionnaire were tested by several researchers not related to this study. All participants were offered a 10,- gift card. Data were collected from June until October 2019 and were stored on a password protected user account.

### Attitudes towards deprescribing

The rPATD questionnaire consists of two global statements and four factors each containing five statements regarding medication and deprescribing of medication in general. Each statement can be answered on a 5-point Likert scale ranging from strongly disagree to strongly agree. The global statements refer to overall satisfaction about medication: ‘Overall, I’m satisfied with my current medicines’ and willingness to stop medication: ‘If my doctor said it was possible, I would be willing to stop one or more of my regular medicines’. The four factors cover ‘burden’, ‘appropriateness’, ‘concerns about stopping’ and ‘involvement in decision making’ in relation to deprescribing [49]. For the medication specific part, the items related to “appropriateness’ and ‘concerns about stopping’ were adapted by replacing the word “medication(s)” with either “insulin”, “antihypertensive medication”, “cholesterol lowering drug” or a specific generic name of the sulfonylurea the participant was taking. For example, “I would like to try stopping the insulin I use to see how I feel without it” in the appropriateness factor. The specific generic name of sulfonylureas was provided because it was anticipated that many participants would not be able to recognize sulfonylureas as a medication group. It was explained that diuretics or “water tablets” were also considered to be antihypertensive medication.

### Determinants

The patients’ age, sex, and the number of medications used (1–5, 6–10 or more than 10 medications) were self-reported. Information on the therapeutic area was derived from the medication dispensed by the pharmacists and self-report. Two groups were distinguished, that is, patients receiving sulfonylurea and/or insulin with or without cardiovascular medication (diabetes area) and patients receiving a statin and at least one antihypertensive medication but no insulin or sulfonylurea (cardiovascular area).

### Analyses

Participants were excluded from analyses when less than 50% of the total rPATD questions were completed. For each of the four factors from the rPATD the average of the five statements was calculated. The answers of the appropriateness factor were scored inversely to obtain the factor score [49]. Although the factor scores of the rPATD have no official cut-off points, we have categorised the average factor scores into disagree (1–2.5), neutral (2.6–3.5) and agree (3.6–5.0) for the descriptive statistics, as previously proposed [48]. Linear regression was used to assess the influence of the age, sex, therapeutic area and number of medications on appropriateness, concerns about stopping, involvement and burden. The answer of the two global questions were categorised into agree, neutral and disagree and ordinal logistic regression was used to assess the influence of the age, sex, therapeutic area and number of medications.

T-tests or Wilcoxon rank-sum tests depending on the normality of the data were used to assess differences in ‘appropriateness’ and ‘concerns about stopping’ between medication groups. Paired tests were used for within-participant comparisons between attitudes towards antihypertensive medication and statins. Unpaired tests were used for between-participant comparisons of attitudes towards the other medication groups. For the comparison between insulins and sulfonylureas, when participants completed the rPATD for both insulin and sulfonylurea, only the insulin scores were used to prevent a mixture of within and between participant comparisons. Chi-squared tests and unpaired t-test were used to compare participants that completed the online versus the paper survey on age, sex and number of medications. Cohen’s d was used to calculate effect sizes of differences between the medication groups. Bonferonni corrections were used to correct for multiple testing. Stata® version 14.2 was used to analyse the data.

### Compliance with ethical standards

The Medical Ethics Review Board of the University Medical Center Groningen concluded that the study did not require a Research Involving Human Subjects Act (WMO) approval because it is not a clinical research with human participants as meant in the Medical Research Involving Human Subjects Act.

## Results

In total 1143 people were invited to participate in the study of which 349 responded. After exclusion of 35 participants who had completed less than 50% of the questions, 314 participants were included resulting in a response rate of 27.5%. Of these, 265 completed the questionnaire online and 49 completed the paper-based questionnaire. Participants who completed the paper questionnaire were on average about 5 years older (*P* < 0.0001), were more often female (*p* = 0.001) and did not use a different number of medications (*P* = 0.191) compared to the participants who completed the online questionnaire. In total 91 participants completed the specific part on a sulfonylurea and/or insulin and 223 participants completed the specific part on a statin and antihypertensive medication. The median age was 76 years, 54% were female, and 52% used more than 5 medications (Table 1). Most participants were satisfied with their current medication (80%). Willingness to stop one or more medications if their doctor said it was possible was also common (88%). In general, few participants (9%) seemed to be burdened by their medication or perceived their medication as not appropriate (12%). On the other hand, few participants (7%) seemed to have concerns towards deprescribing medication. Most participants (85%) would like to be involved in medication decisions. Some opposing attitudes were observed when looking at individual items (Table 2). Although more than a third of the participants (36%) felt that they were taking a large number of medications, only a small proportion (9%) had a score above 3.5 on the burden factor. Also, despite the small number of participants with concerns about stopping medication, 55% of the participants would be reluctant to stop medication that they were taking for a long period and 36% would be worried about missing out on future benefits if one of their medicines was stopped.

**Table 1:** Patients characteristics and scores on their general attitudes towards deprescribing subdivided by therapeutic area

	Total	Diabetes area^a^	Cardiovascular area^a^
**Number of participants**	314	91	223
**Age (years), median (IQR)**	76 (IQR:73–80)	76 (IQR:73–80)	75 (IQR:73–80)
**Sex, n (%)**			
Female	118 (38%)	34 (37%)	84 (38%)
Male	192 (61%)	57 (63%)	135 (61%)
Unknown	4 (1%)	0 (0%)	4 (2%)
**Number of medication, n (%)**			
1–5	151 (48%)	35 (38%)	116 (52%)
6–10	137 (44%)	43 (47%)	94 (42%)
> 10	26 (8%)	13 (14%)	13 (6%)
**Satisfaction with medication**^b^			
Agree, n (%)	250 (80%)	76 (84%)	174 (78%)
Neutral, n (%)	45 (14%)	8 (9%)	37 (17%)
Disagree, n (%)	19 (6%)	7 (8%)	12 (5%)
**Willingness to stop**^c^			
Agree, n (%)	276 (88%)	83 (91%)	193 (87%)
Neutral, n (%)	22 (7%)	5 (5%)	17 (8%)
Disagree, n (%)	16 (5%)	3 (3%)	13 (6%)
**Burden**			
Mean (SD)	2.6 (0.62)	2.7 (0.69)	2.6 (0.58)
Agree, n (%)	29 (9%)	15 (17%)	14 (6%)
Neutral, n (%)	150 (48%)	38 (42%)	112 (50%)
Disagree, n (%)	134 (43%)	37 (41%)	97 (44%)
**Appropriateness**			
Mean (SD)	3.7 (0.65)	3.6 (0.74)	3.7 (0.60)
Agree, n (%)	128 (41%)	39 (43%)	89 (40%)
Neutral, n (%)	148 (47%)	41 (45%)	107 (48%)
Disagree, n (%)	36 (12%)	11 (12%)	25 (11%)
**Concerns**			
Mean (SD)	2.7 (0.62)	2.7 (0.67)	2.7 (0.59)
Agree, n (%)	22 (7%)	6 (7%)	16 (7%)
Neutral, n (%)	165 (53%)	49 (54%)	116 (53%)
Disagree, n (%)	123 (40%)	35 (39%)	88 (40%)
**Involvement**			
Mean (SD)	3.9 (0.60)	3.9 (0.59)	4.0 (0.60)
Agree, n (%)	266 (85%)	75 (82%)	191 (86%)
Neutral, n (%)	35 (11%)	12 (13%)	23 (10%)
Disagree, n (%)	11 (4%)	4 (4%)	7 (3%)

The factor scores are reported as the mean (SD) of five Likert scale items and are categorised into disagree (1–2.5), neutral (2.6–3.5) and agree (3.6–5.0)

^a^In the diabetes area group, 72 (79%) participants reported that they were using a antihypertensive medication, 65 (71%) reported using a statin, 64 (70%) used a sulfonylurea and 33 (36%) used insulin. In the cardiovascular area group, all participants used at least one antihypertensive medication, a statin and no sulfonylurea or insulin

^b^Satisfaction with medication: ‘Overall, I’m satisfied with my current medicines’

^c^Willingness to stop: ‘If my doctor said it was possible, I would be willing to stop one or more of my regular medicines’

**Table 2:** Patients’ responses to individual items of the general revised Patients’ Attitudes Towards Deprescribing (rPATD)

Item	Disagree & strongly disagree (%)	Unsure (%)	Strongly agree & agree (%)
**Appropriateness (scores are not reversed for individual items)**			
**A1:** I would like to try stopping one of my medicine to see how I feel without it *(n = 314)*	37	34	29
**A2:** I would like my doctor to reduce the dose of one or more of my medicine *(n = 313)*	33	41	26
**A3:** I feel that I may be taking one or more medicines that I no longer need *(n = 313)*	44	39	17
**A4:** I believe one or more of my medicines may be currently giving me side effects *(n = 313)*	58	19	23
**A5:** I think one or more of my medicines may not be working *(n = 313)*	64	31	5.4
**Burden**			
**B1:** I feel that I am taking a large number of medicines *(n = 313)*	22	42	36
**B2:** Taking my medicines every day is very inconvenient *(n = 314)*	70	21	8.3
**B3:** I spend a lot of money on my medicines *(n = 314)*	39	35	25
**B4:** Sometimes I think I take too many medicines *(n = 314)*	43	38	19
**B5:** I feel that my medicines are a burden to me *(n = 314)*	66	25	10
**Concerns about stopping**			
**C1:** I have bad experience when stopping a medicine before *(n = 312)*	58	27	15
**C2:** I would be reluctant to stop a medicine that I had been taking for a long time *(n = 313)*	21	24	55
**C3:** If one of my medicines was stopped I would be worried about missing out on future benefits *(n = 313)*	28	35	36
**C4:** I get stressed whenever changes are made to my medicines *(n = 313)*	60	25	14
**C5:** If my doctor recommended stopping a medicine I would feel that he/she was giving up on me *(n = 312)*	77	16	7.4
**Involvement**			
**I1:** I like to be involved in making decisions about my medicines with my doctors *(n = 314)*	4.8	13	82
**I2:** I have a good understanding of the reasons I was prescribed each of my medicines *(n = 314)*	6.1	10	84
**I3:** I like to know as much as possible about my medicines *(n = 314)*	6.1	30	64
**I4:** I always ask my doctor, pharmacist or other health care professional if there is something I don’t understand about my medicines *(n = 313)*	6.7	12	81
**I5:** I know exactly what medicines I am currently taking, and/or I keep an up to date list of my medicines *(n = 313)*	5.1	6.1	89

### Influence of patient characteristics on attitudes towards deprescribing

Age, sex, therapeutic area or number of medications were not associated with the willingness to stop medication and the satisfaction with their current medication (Table 3). All participants with more than ten medications were willing to stop medication and therefore a reliable estimate of odds ratio was mathematically not possible. A Fischer’s exact test showed that there was no association between taking more than ten medications and willingness to stop (*p* = 0.22). No significant differences were found for the appropriateness, concerns about stopping and involvement factors for any of the explanatory characteristics. Taking more than ten medicines was significantly associated with a higher medication burden.

**Table 3:** Association between participants’ demographics and the responses on the two global questions and the four factor scores of the revised Patients’ Attitudes Towards Deprescribing (rPATD)

		Satisfaction with medication^a^ (*n* = 310)		Willingness to stop^a^ (*n* = 310)		Burden^b^ (*n* = 309)		Appropriateness^b^ (*n* = 308)		Concerns about stopping^b^ (*n* = 306)		Involvement^b^ (*n* = 308)	
		OR	*p*	OR	*p*	Coefficient	*p*	Coefficient	*p*	Coefficient	*p*	Coefficient	*p*
**Age** (years)		1.0	*0.15*	1.0	*0.92*	−0.005	*0.50*	−0.008	0.31	0.005	0.50	−0.005	0.48
**Sex (**male)		0.75	*0.32*	0.88	*0.72*	−0.007	*0.80*	−0.093	0.23	0.075	0.31	0.085	0.23
**Number of medicines**	(≤5)	0.61	*0.12*	0.87	*0.72*	0.018	*0.35*	0.098	0.22	0.074	0.34	0.040	0.58
	(> 10)	0.61	*0.36*	–	*–*	0.41	0.002**	0.15	0.30	0.080	0.55	−0.047	0.72
**Therapeutic area**		0.76	*0.40*	0.71	*0.41*	−0.097	*0.21*	0.086	0.30	−0.022	0.78	0.071	0.35
**Total model**			*0.15*		*0.16*		0.004**		0.28		0.80		0.56

^a^Ordinal logistic regression model

^b^Linear regression model

**Significant *P*-value after correcting for multiple testing (*p* = 0.0083): participants with more than 10 medicines scored higher on the burden factor

- All 25 participant with > 10 medicines agreed with the statement, therefore no reliable estimation could be made

Therapeutic area = Comparing patients using sulfonylurea and/or insulin (=0) and patient using a statin and at least one antihypertensive medication but no insulin or sulfonylurea (=1)

### Attitudes towards deprescribing comparing cardiometabolic medications

Both antihypertensive medication and insulins were considered more appropriate in comparison to statins not favouring deprescribing (Table 4). Additionally, insulins were considered more appropriate than sulfonylureas but this difference was not significant after correcting for multiple testing. There were no significant differences in concerns about stopping medication between insulins, sulfonylureas, antihypertensive medication and statins.

**Table 4:** Comparison of the perceived appropriateness and the concerns about stopping of insulin, sulfonylurea, antihypertensive and statin

	**Number of participants (n)**	**Mean**	**SD**	***P*** **value**	**Effect size** (**Cohen’s D)**
**Appropriateness of the medication**^**d**^					
Insulin^a^	32	3.81	0.84	0.026*	0.51
Sulfonylurea^e^	50	3.43	0.68		
Insulin^a^	32	3.81	0.84	0.002**	0.59
Statins	209	3.38	0.71		
Sulfonylureas^a^	64	3.56	0.75	0.20	0.21
Antihypertensive	182	3.69	0.61		
Statin^b^	170	3.38	0.69	< 0.001**	0.47
Antihypertensive	170	3.68	0.63		
	**(n)**	**Median**	**IQR**	***P*** **value**	**Effect size**
Insulin^c^	32	3.8	2.2–3.4	0.53	0.19
Antihypertensive	182	3.8	2.4–3.0		
Sulfonylurea^c^	64	3.6	2.0–3.0	0.090	0.24
Statin	209	3.4	1.8–3.0		
**Concerns about stopping medication**					
	**(n)**	**Mean**	**SD**	***P*** **value**	**Effect size**
Insulin^a^	31	2.61	0.83	0.88	0.040
Sulfonylurea	46	2.59	0.51		
Insulin^a^	32	2.63	0.82	0.86	0.034
Antihypertensive	183	2.65	0.64		
Insulin ^a^	32	2.63	0.82	0.86	0.041
Statin	204	2.60	0.60		
Sulfonylurea ^a^	58	2.59	0.60	0.52	0.097
Antihypertensive	183	2.65	0.64		
Sulfonylurea^a^	58	2.59	0.62	0.89	0.021
Statin	204	2.60	0.60		
Statin ^b^	169	2.57	0.58	0.19	0.079
Antihypertensive	169	2.63	0.65		

^a^Unpaired student t-test, between participants comparison

^b^Paired student t-test, within participant comparison

^c^Wilcoxon rank-sum, between participant comparison

^d^Questions of appropriateness are scored inversely to obtain the factor score (means and median), a higher appropriateness score thus means that participant considered their medication more appropriate

^e^When comparing insulin to sulfonylureas there were 14 participants who used both medication groups. For the comparison for these participants only the insulin answers were used to prevent a mixture of within and between participants comparison

*p-value of ≤0.05 is considered significant before correction for multiple testing

**p-value of ≤0.0083 is considered significant after correction for multiple testing

Looking at the items underlying the main factors of the rPATD questionnaire, although no statistical testing was done, some variations are of note (Supplementary tables, tables S1, S2, S3 and S4). It seems that fewer participants wanted to try to stop their insulin (12%), antihypertensive (12%) or sulfonylurea (19%) than their statin (32%) or one of their medicines in general (29%) (Table 2). Conversely, less participants were reluctant to stop their statin (38%) or sulfonylurea (38%) than their antihypertensive (46%), insulin (52%) or one of their medicines in general (55%). Furthermore, there were less participants who wanted their doctor to reduce the dose of their insulin (15%) or their antihypertensive (11%) compared to participants who wanted to reduce the dose of their statin (22%), sulfonylurea (20%) or one or more of their medicines in general (26%).

## Discussion

The studied patient characteristics did not influence most of the attitudes towards deprescribing, except for perceived burden of medication which was higher among patients using more than ten chronic medications. Significant differences were observed towards the perceived appropriateness of specific cardiometabolic medications in relation to deprescribing but no differences were found in the patients’ general concerns about stopping these specific medications. There were indications of relevant differences in specific underlying attitudes, such as the reluctance to stop specific medication.

### Comparison with literature

#### Influence of patients’ demographics on general attitudes towards deprescribing

This study confirmed findings from previous studies that most older people are satisfied with their current medication but at the same time are willing to stop medication if their doctor said it was possible [21, 22, 27–36, 52]. Willingness to stop and satisfaction with current medication were found to be common regardless of the patients’ age, sex or therapeutic area. The lack of association between these characteristics and general attitudes towards deprescribing is in contrast to findings from a study in Malaysia, where several associations were found with age [30]. Part of this might be explained by the studied population which we restricted to older people using cardiometabolic medication. In our study, taking more than ten medications was related to a higher perceived burden but also it did not significantly increased willingness to stop medication. Previous findings from the US are inconsistent, with one study showing patients that took six or more medications to be more often willing to stop while another study found no correlation [36, 45]. Finally, a Swiss study also showed no association between sex, age or the number of medications and willingness to stop medication [27]. In this Swiss study, however, it was observed that participants with a higher education and a good relationship with their general practitioner were more willing to consider stopping [27]. Polypharmacy is consistently found to be associated with a higher burden [30, 45]. The lack of association between the therapeutic area and the attitudes towards deprescribing may be caused by the fact that most patients in the diabetes area group also used cardiovascular medication.

#### Attitudes towards deprescribing of specific medication groups

Although patients might be willing to stop medication in general, they might not be willing to stop medication they consider essential [44]. A study conducted in Australia illustrated that older people were more willing to stop taking their statin than to stop one or more of their medication in general when their doctor would say this was possible [29]. The perceived appropriateness of specific medication could be an important factor for willingness to undergo deprescribing in practice, since a previous study showed that two of the appropriateness items significantly predicted willingness to stop [21].

In our study we observed that insulins were considered more appropriate than statins in relation to deprescribing. This difference might be caused by differences in the subpopulations. The general patient characteristics included in our study, however, were not associated with the general appropriateness factor. The higher perceived appropriateness of insulin might be caused by the efforts of health care providers that are often needed to convince type 2 diabetes patients to start injecting insulin. Although health care providers may stress the need for any newly initiated medication, this is particularly relevant to counter initial resistance to insulin. In type 2 diabetes patients there is a high initial resistance to start with insulin [53, 54]. Some patients have a fear for needles or fear weight gain and hypoglycaemia [53, 55, 56]. To counter initial resistance, health care providers often stress the need for insulin to control glucose and to prevent complications [53]. The higher perceived appropriateness of antihypertensive medication compared to statins might be explained by differences in perceived severity of the underlying disease. One could expect that when patients perceive hypertension as more severe than hyperlipidaemia, they perceive antihypertensive medication as more appropriate or needed in comparison to statins. Such a difference in perceived disease severity was observed in a qualitative study among African-American men who perceived hypertension to be more severe than hyperlipidaemia [57]. In line with this, patients in a Dutch survey study considered statins to be of low importance, while anticoagulants, glucose lowering medication and antihypertensive medication were considered highly important [58]. Although it is likely that medication that is perceived to be appropriate and that is taken for a disease that is perceived to be severe will result in a high perceived importance, further research is needed to establish whether these concepts are linked this way by patients. The lower scores in perceived appropriateness for statins might in part be fuelled by negative media attention towards statins and the higher perceived sided effects of statins. Danish and British research showed that discontinuation of statins increased after negative media attention particularly among patients with less severe disease [59, 60].

The results of this study show that the medication appropriateness of the general rPATD is similar to the appropriateness of statins, the medication group with the lowest scores for appropriateness. This indicates that when participants complete the general appropriateness items they think of the medication they consider to be the least appropriate. Therefore patients who score relatively low on the appropriateness factor in general might only consider one of their medications to be inappropriate and would therefore only be willing to stop that medication. In a study by Edelman et al. (2019), the appropriateness of alpha-blockers was higher than the general appropriateness [48]. This supports the notion that attitudes towards deprescribing of specific drugs should not be derived from a patient’s general attitudes. Further research is needed comparing multiple medication groups with medication in general to explore how these concepts of perceived appropriateness are linked. We observed that concerns about stopping were higher for specific medications compared to the general rPATD questions. These results suggest that when completing the items related to the concerns factor, participants might not always think of the medication that they would be most concerned to stop. This might result in an underestimation of patient’s concerns about stopping medication when using the general rPATD.

Surprisingly, there were no differences in concerns about stopping between insulins, sulfonylureas, antihypertensive medication and statins. The compared medication groups were all long-term preventive medication for either type 2 diabetes or cardiovascular diseases. Concerns about stopping might be different for symptomatic relief medication, short term medication or medication used for other diseases that are considered more or less severe [41]. Still, the results of this study might be an indication that the concerns factor is not discriminative to detect differences. Age, sex, therapeutic area, and number of medications also did not affect concerns about stopping. Other personal or health care related factors unrelated to the medication group may still affect concerns about stopping like the relationship with the physician, educational level, health literacy or a general dislike for medication [30, 41, 42].

### Strengths and limitations

This is a first study assessing attitudes towards deprescribing across different medication groups. A linguistically validated questionnaire was used in a relatively large sample of older Dutch patients recruited from five different locations across the Netherlands. The most important limitation, common for survey studies, is a low response rate. The influence of non-response rate bias is hard to test but has found to be small in other survey studies [61, 62]. Selection bias can be introduced due to the topic of the survey. Patients that are more interested in stopping medication and are more involved in their medication might be more likely to complete the survey. This could result in an overestimation of the involvement score. The generalizability is reduced by excluding participants unable to read and write in Dutch. This is likely to result in an underrepresentation of minority groups and patients with low health literacy. For the comparison of the medication groups a mix of within and between participant’s designs were used. Differences in attitudes found in the between person analyses might be caused by differences in the population instead by differences caused by the medication groups. We did not distinguish between different antihypertensive medication groups, patients that took multiple antihypertensive medications answered the questions for these medication as a group, which might not reflect how they think about these medications. Lastly, we only included demographic and treatment characteristics that are easily accessible to health care providers in our analyses to determine which characteristics influence attitudes towards deprescribing. However, this provides an incomplete picture of which patients’ characteristics may influence attitudes towards deprescribing.

### Implications for practice and research

Previous research has already established that older people want to be involved in decisions about changing their medication, and this is also the case for older people with type 2 diabetes or cardiovascular diseases [21–23]. When health care providers want to discuss deprescribing cardiometabolic medication, it is important to address the perceived appropriateness of the specific medication. Even more so because willingness to deprescribe may not be associated with actual potential inappropriate medication use, indicating that patients are not necessarily aware of which medication is inappropriate [28]. Particularly, an explanation may be needed why medication that was appropriate for the patient in the past, is no longer required or can even be harmful. Many of the same beliefs about medication addressed by physicians to support good adherence can influence willingness to stop medication. This needs to be taken into account when medication is started or intensified. This can be done by communicating already at the start of medication that the medication has to be taken for a long time, but that periodically re-evaluating is needed. This can facilitate deprescribing efforts in the future.

Although the participants using more than ten different medications appeared to be just as satisfied with their medication than those using less, they did perceive a higher burden from the medication and all of them were willing to stop medication. Targeting older patients with a large number of medications for deprescribing interventions could help relieve this burden. An Irish study, however, did not show a reduction in the rPATDs burden factor after a medication review was conducted in a group of 54 older patients, showing that reducing medication burden might be difficult to achieve [63].

In this study, we investigated the association between commonly available patients’ characteristics and general attitudes towards deprescribing and found that such general factors are of limited value to tailor conversations about deprescribing. Future research could focus on other patients’ characteristics that may influence attitudes towards deprescribing, for example, health literacy, relationship with the health care provider, social support, and illness beliefs or awareness, preferably using instruments that can be easily applied in clinical practice. Also, more attention is needed for medication-specific attitudes towards deprescribing.

## Conclusion

The majority of older people using cardiometabolic medication are willing to stop medicines if their doctor said it was possible but may be reluctant to stop a specific medication. Health care providers should take into account that patients perceive some of their medication as more appropriate than other medication when discussing deprescribing. Regarding cardiometabolic medication, such as included in our study, patients considered insulins and antihypertensive medication more appropriate than sulfonylureas and statins.

## Supplementary Information

**Additional file 1: Table S1.** Appropriateness and concerns about stopping sulfonylureas revised Patients’ Attitudes Towards Deprescribing **(**rPATD) questions. **Table S2.** Appropriateness and concerns about stopping insulin revised Patients’ Attitudes Towards Deprescribing **(**rPATD) questions. **Table S3.** Appropriateness and concerns about stopping antihypertensive medication revised Patients’ Attitudes Towards Deprescribing **(**rPATD) questions. **Table S4.** Appropriateness and concerns about stopping statin revised Patients’ Attitudes Towards Deprescribing **(**rPATD) questions.

## Data Availability

The datasets generated and analysed during the current study are not publicly available as this would be in conflict with the informed consent given by the participants, but are available from the corresponding author on reasonable request. Raw data is not available as this would be in conflict with the informed consent given by the participants.

## Abbreviations

rPATD: Revised Patients’ Attitudes Towards Deprescribing
US: United States
ATC: Anatomical Therapeutic Chemical
