# Supplementary material for: Older people’s attitudes towards deprescribing cardiometabolic medication
Source: BMC Geriatr. 2021 Jun 16;21:366. doi: 10.1186/s12877-021-02249-z (PMC8207766; doi:10.1186/s12877-021-02249-z)
Supplement: Supplementary file 1 — Additional file 1: Table S1. Appropriateness and concerns about stopping sulfonylureas revised Patients’ Attitudes Towards Deprescribing (rPATD) questions. Table S2. Appropriateness and concerns about stopping insulin revised Patients’ Attitudes Towards Deprescribing (rPATD) questions. Table S3. Appropriateness and concerns about stopping antihypertensive medication revised Patients’ Attitudes Towards Deprescribing (rPATD) questions. Table S4. Appropriateness and concerns about stopping statin revised Patients’ Attitudes Towards Deprescribing (rPATD) questions. [file 12877_2021_2249_MOESM1_ESM.docx]

# Older people’s attitudes towards deprescribing cardiometabolic medication

*Stijn Crutzen^1^, Jamila Abou^2^, Sanne E Smits^1^, Gert Baas^3^, Jacqueline G Hugtenburg^2^, Mette Heringa^3,4^, Petra Denig^1^ and Katja Taxis^5^.*

1 Department of Clinical Pharmacy and Pharmacology, University Medical Center Groningen, University of Groningen, Groningen, Netherlands
2 Department of Clinical Pharmacology and Pharmacy, Amsterdam UMC, location VUMC, Amsterdam, The Netherlands
3 SIR Institute for Pharmacy Practice and Policy, Theda Mansholtstraat 5B, 2331 JE, Leiden, The Netherlands
4 Division of Pharmacoepidemiology and Clinical Pharmacology, Utrecht Institute for Pharmaceutical Sciences, Utrecht University, Utrecht, The Netherlands
5 Unit of PharmacoTherapy, Epidemiology and Economics, Groningen Research Institute of Pharmacy, University of Groningen, Groningen, The Netherlands

Corresponding author: Stijn Crutzen: [s.crutzen@umcg.nl](mailto:s.crutzen@umcg.nl)

Universitair Medisch Centrum Groningen
Stijn Crutzen Clinical Pharmacy and Pharmacology
EB70
Postbus 30.001
Hanzeplein1
9700 RB Groningen, The Netherlands

Supplementary tables

Table S1. Appropriateness and concerns about stopping sulfonylureas revised Patients’ Attitudes Towards Deprescribing **(**rPATD) questions

| **Item** | **Disagree & strongly disagree (%)** | **Unsure (%)** | **Strongly agree & agree (%)** |
| --- | --- | --- | --- |
| **Appropriateness** | | | |
| I would like to try stopping the sulfonylurea to see how I feel without it *(n=64)* | 48 | 33 | 19 |
| I would like my doctor to reduce the dose of the sulfonylurea *(n=64)* | 48 | 31 | 20 |
| I feel that I may be taking the sulfonylurea that I no longer need *(n=64)* | 56 | 38 | 6.3 |
| I believe the sulfonylurea may be currently giving me side effects *(n=64)* | 63 | 33 | 4.7 |
| I think the sulfonylurea may currently not be working *(n=64)* | 59 | 39 | 1.6 |
| **Concerns about stopping** | | | |
| I have had a bad experience when stopping the sulfonylurea before *(n=64)* | 47 | 45 | 7.8 |
| I would be reluctant to stop the sulfonylurea that I had been taking for a long time *(n=64)* | 25 | 38 | 38 |
| If the sulfonylurea was stopped I would be worried about missing out on future benefits *(n=64)* | 36 | 36 | 28 |
| I get stressed whenever changes are made to my sulfonylurea *(n=64)* | 63 | 36 | 1.6 |
| If my doctor recommended stopping the sulfonylurea I would feel that he/she was giving up on me *(n=64)* | 64 | 30 | 6.3 |

Table S2. Appropriateness and concerns about stopping insulin revised Patients’ Attitudes Towards Deprescribing **(**rPATD) questions

| **Item** | **Disagree (%)** | **Neutral (%)** | **Agree (%)** |
| --- | --- | --- | --- |
| **Appropriateness** | | | |
| I would like to try stopping the insulin to see how I feel without it *(n=33)* | 64 | 24 | 12 |
| I would like my doctor to reduce the dose of the insulin *(n=33)* | 48 | 36 | 15 |
| I feel that I may be taking the insulin that I no longer need *(n=33)* | 73 | 24 | 3.0 |
| I believe the insulin may be currently giving me side effects *(n=32)* | 69 | 25 | 6.3 |
| I think the insulin may currently not be working *(n=32)* | 84 | 13 | 3.1 |
| **Concerns about stopping** | | | |
| I have had a bad experience when stopping the insulin before *(n=32)* | 56 | 41 | 3.1 |
| I would be reluctant to stop the insulin that I had been taking for a long time *(n=33)* | 33 | 15 | 52 |
| If the insulin was stopped I would be worried about missing out on future benefits *(n=33)* | 30 | 24 | 45 |
| I get stressed whenever changes are made to my insulin *(n=33)* | 61 | 33 | 6.1 |
| If my doctor recommended stopping the insulin I would feel that he/she was giving up on me *(n=33)* | 58 | 24 | 18 |

Table S3. Appropriateness and concerns about stopping antihypertensive medication revised Patients’ Attitudes Towards Deprescribing **(**rPATD) questions

| **Item** | **Disagree (%)** | **Neutral (%)** | **Agree (%)** |
| --- | --- | --- | --- |
| **Appropriateness** | | | |
| I would like to try stopping the antihypertensive medication to see how I feel without it *(n=185)* | 59 | 29 | 12 |
| I would like my doctor to reduce the dose of the antihypertensive medication *(n=184)* | 53 | 36 | 11 |
| I feel that I may be taking the antihypertensive medication that I no longer need *(n=185)* | 64 | 31 | 5.4 |
| I believe the antihypertensive medication may be currently giving me side effects *(n=183)* | 72 | 22 | 5.5 |
| I think the antihypertensive medication may currently not be working *(n=185)* | 76 | 22 | 2.2 |
| **Concerns about stopping** | | | |
| I have had a bad experience when stopping the antihypertensive medication before *(n=185)* | 61 | 31 | 8.7 |
| I would be reluctant to stop the antihypertensive medication that I had been taking for a long time *(n=184)* | 28 | 26 | 46 |
| If the antihypertensive medication was stopped I would be worried about missing out on future benefits *(n=184)* | 28 | 32 | 40 |
| I get stressed whenever changes are made to my antihypertensive medication *(n=184)* | 56 | 30 | 14 |
| If my doctor recommended stopping the antihypertensive medication I would feel that he/she was giving up on me *(n=184)* | 76 | 16 | 8.7 |

Table S4. Appropriateness and concerns about stopping statin revised Patients’ Attitudes Towards Deprescribing **(**rPATD) questions

| **Item** | **Disagree (%)** | **Neutral (%)** | **Agree (%)** |
| --- | --- | --- | --- |
| **Appropriateness** | | | |
| I would like to try stopping the statin to see how I feel without it *(n=209)* | 38 | 30 | 32 |
| I would like my doctor to reduce the dose of the statin *(n=209)* | 36 | 41 | 22 |
| I feel that I may be taking the statin that I no longer need *(n=209)* | 42 | 45 | 14 |
| I believe the statin may be currently giving me side effects *(n=209)* | 61 | 20 | 19 |
| I think the statin may currently not be working *(n=209)* | 60 | 38 | 2.4 |
| **Concerns about stopping** | | | |
| I have had a bad experience when stopping the statin before *(n=207)* | 64 | 32 | 3.9 |
| I would be reluctant to stop the statin that I had been taking for a long time *(n=207)* | 28 | 34 | 38 |
| If the statin was stopped I would be worried about missing out on future benefits *(n=207)* | 26 | 34 | 40 |
| I get stressed whenever changes are made to my statin *(n=206)* | 62 | 27 | 11 |
| If my doctor recommended stopping the statin I would feel that he/she was giving up on me *(n=206)* | 74 | 15 | 11 |
